# Patients with MAC Lung Disease Have a Low Visceral Fat Area and Low Nutrient Intake

**DOI:** 10.1155/2015/218253

**Published:** 2015-11-29

**Authors:** Kentaro Wakamatsu, Nobuhiko Nagata, Sanae Maki, Hisamitsu Omori, Hiroyuki Kumazoe, Kayoko Ueno, Yuko Matsunaga, Makiko Hara, Koji Takakura, Nagisa Fukumoto, Nobuhisa Ando, Mami Morishige, Takashi Akasaki, Ichiro Inoshima, Shinji Ise, Miiru Izumi, Masayuki Kawasaki

**Affiliations:** ^1^Department of Respiratory Medicine, National Hospital Organization, Omuta Hospital, 1044-1 Tachibana, Omuta 837-0911, Japan; ^2^Department of Respiratory Medicine, Fukuoka University Chikushi Hospital, Japan; ^3^Faculty of Life Sciences, Kumamoto University, Japan; ^4^Department of Nutrition, National Hospital Organization Omuta National Hospital, Japan

## Abstract

*Objective.* This study aimed to examine the nutritional status and nutrient intake of patients with MAC lung disease with a focus on visceral fat area.* Patients and Methods.* Among 116 patients of our hospital with nontuberculous mycobacteriosis who were registered between May 2010 and August 2011, 103 patients with MAC lung disease were included in this study. In all patients, nutritional status and nutrient intake were prospectively examined.* Results.* Patients were 23 men and 80 women (mean age, 72.3 ± 10.9 years). BMI (kg/m^2^) at the time of registration was 20.4 ± 2.7 in men and 19.2 ± 2.9 in women. Visceral fat area (cm^2^) was significantly lower in women (35.7 ± 26.6) than in men (57.5 ± 47.4) (*p* = 0.0111). The comparison with general healthy adults according to age revealed a markedly reduced visceral fat area among patients with MAC lung disease. With respect to nutrient intake, energy adequacy (86.1 ± 15.7%), protein adequacy (82.4 ± 18.2%), lipid adequacy (78.1 ± 21.8%), and carbohydrate adequacy (89.6 ± 19.2%) ratios were all low at the time of registration. BMI was significantly correlated with protein adequacy (*p* = 0.0397) and lipid adequacy (*p* = 0.0214) ratios, while no association was found between visceral fat area and nutrient intake.* Conclusion.* Patients with MAC lung disease had a low visceral fat area and low nutrient intake.

## 1. Introduction

Mycobacterium avium complex (MAC) lung disease is often observed in slim middle-aged and older women [1–4], and slenderness has been reported to serve as a prognostic factor [4, 5]. Although it is unclear how weight loss is involved with the etiology and pathology of MAC lung disease, the abnormal production of inflammatory cytokines due to fluctuations in adipocyte-derived adipokines, which is caused by a decrease in fat cells (particularly visceral fat cells) due to weight loss, has been suggested as one of the contributory factors [6]. In this context, it will be meaningful to investigate the association between weight loss and visceral fat area in patients with MAC lung disease, but no report exists with regard to weight loss and visceral fat area in patients with MAC lung disease. Moreover, while it is predicted that weight loss in patients with MAC lung disease is attributable to emaciation from the illness itself as well as low nutrient intake, no published reports pertaining to nutrient intake exist. Accordingly, this study aimed to examine the nutritional status and nutrient intake of patients with MAC lung disease with a focus on visceral fat area.

## 2. Patients and Methods

At our hospital, 116 outpatients and inpatients with NTM disease were registered between May 2010 and September 2013. Among these, 103 patients with MAC lung disease were included in this study. All patients met the diagnostic criteria of “An Official ATS/IDSA Statement: Diagnosis, Treatment, and Prevention of Nontuberculous Mycobacterial Diseases” [7] based on imaging and bacteriological examination findings, agreed with the study content, and provided written consent to participate in this study. We first compared nutritional status and nutritional intake between FC and NB dis. and found no differences between them. Accordingly, these two groups were combined.

Patients currently under treatment were defined as those undergoing a combination therapy that included clarithromycin or new quinolone antibacterial agents. Assessment of nutritional status included body mass index (BMI), lymphocyte count, serum albumin, serum prealbumin, serum cholinesterase, serum transferrin, total cholesterol, visceral fat area assessed by abdominal CT, and waist circumference. Measurements of visceral fat area and waist circumference were obtained from abdominal CT scans taken at the level of umbilicus using image analysis software (Fat Pointer; version 1.10, Hitachi Medical Corporation, Tokyo, Japan). Furthermore, BMI, waist circumference, and visceral fat area were compared with data of general adult participants of health checkups in 2008 FY (649 patients; 418 men, 231 women) from the Japanese Red Cross Kumamoto Hospital.

Information regarding dietary content and intake was obtained through interviews by nutritionists. Preliminarily, dietary and intake survey by nutritionists was done twice during a month period in ten patients with MAC lung disease, respectively, and we confirmed the reproducibility of results of the survey. Calorie intake, protein, lipid, and carbohydrate adequacy ratios (actual intake amount of each nutrient/average intake among Japanese people determined based on the 2010 National Nutrition Survey × 100) were determined.

Data were expressed as mean ± standard deviation (SD), since a test of normality showed that all data were normally distributed. *t*-tests were used for comparisons with data of general adults regarding visceral fat area. *p* < 0.05 was considered statistically significant.

This study was conducted with the approval from the Ethics Board of the National Hospital Organization Omuta National Hospital.

## 3. Results

### 3.1. Patient Characteristics

Our patients were predominantly female (24 men and 79 women), with an age range of 46–91 years and mean age of 71.3 ± 10.9 years at the time of registration. Disease duration (from the date of confirmed diagnosis to the point of registration) ranged from 0.93 to 311.8 months, with a mean duration of 78.5 ± 65.0 months. Detected bacterial strains were* M. intracellulare* in 59 patients,* M. avium* in 35, and* M. intracellulare* +* M. avium* in 9. Twenty-six patients had fibrocavitary (FC) disease, and 77 patients had nodular bronchiectatic (NB) disease (Table 1).

### 3.2. Nutritional Status of Patients with MAC Lung Disease

In both men and women, BMI, waist circumference, and visceral fat area at registration were significantly lower compared to data of health checkup participants according to age. In particular, visceral fat area was markedly lower than that of health checkup participants (Table 2).

There was no significant difference in BMI between men and women, but women had a significantly lower visceral fat area. With respect to disease type, significantly more men had FC disease. In blood examination, serum prealbumin levels were low in both men (18.4 ± 4.8 mg/dL) and women (16.4 ± 4.4 mg/dL) compared to normal value of Japanese. However serum albumin, cholinesterase, transferrin, and total cholesterol levels were all within the normal ranges (Table 1).

As for associations between visceral fat area and patient characteristics and laboratory findings, BMI, waist circumference, white blood cell count, and lymphocyte count were significantly correlated in men, but only BMI and waist circumference were significantly correlated in women (Table 3).

### 3.3. Nutrient Intake of Patients with MAC Lung Disease

With respect to nutrient intake, energy adequacy (86.1 ± 15.7%), protein adequacy (82.4 ± 18.2%), lipid adequacy (78.1 ± 21.8%), and carbohydrate adequacy (89.6 ± 19.2%) ratios were low at the time of registration (Table 4). BMI was significantly correlated with protein adequacy (*p* = 0.0397) and lipid adequacy (*p* = 0.0214) ratios (Table 5). However visceral fat area was not significantly correlated with intake of each nutrient (Table 6).

## 4. Discussion

One of the clinical characteristics of MAC lung disease is its high prevalence among slim women. Kim et al. performed a prospective examination of 63 patients with NTM lung disease and reported that, compared with the background-matched control group, BMI was significantly lower [1]. Kartalija et al. also reported similar results in a prospective examination of 103 patients with NTM lung disease [2]. Moreover, in Japan, Okumura et al. reported that, in 273 patients with MAC lung disease, two-thirds were female and had a BMI lower than the standard BMI, irrespective of disease type (i.e., FC or NB) [3]. In the present study, most of our patients were women and had a BMI lower than that of general healthy controls. Although it is unclear how weight loss is involved with the etiology and pathology of MAC lung disease, the abnormal production of inflammatory cytokines due to fluctuations in adipocyte-derived adipokines, which is caused by a decrease in fat cells (particularly visceral fat cells) due to weight loss, has been suggested as one of the contributory factors [6]. Moreover, while it is predicted that weight loss in patients with MAC lung disease is attributable to emaciation from the illness itself as well as low nutrient intake, no published reports pertaining to nutrient intake exist. To our knowledge, this study is the first to examine visceral fat area and nutrient intake in patients with MAC lung disease.

In the present study, BMI, waist circumference, and particularly visceral fat area were significantly lower in both men and women compared with general healthy adults. Moreover, in this study, visceral fat area was strongly correlated with BMI and waist circumference, but not with patient characteristics or disease duration. Tasaka et al. showed a negative correlation between adiponectin and BMI and reported that, compared to healthy individuals, MAC patients had significantly higher levels of adiponectin according to weight [8]. The anti-inflammatory activities of adiponectin extend to inhibition of IL-6 production accompanied by induction of the anti-inflammatory cytokines IL-10 and IL-1 receptor antagonist and increased adiponectin leads to increased susceptibility to infections [9]. A decrease in visceral fat is possibly associated with the development of MAC lung disease, because Staiger et al. reported that a strong association exists between adiponectin and visceral fat [10].

That many patients with MAC lung disease are slim has been consistently shown as well as our patients; however, it is unclear why patients with NTM disease tend to be slim. The present study provides some insight in this regard, as it clearly showed that patients with MAC lung disease have low energy, protein, fat, and carbohydrate intake, which could partially explain why these patients are slim. BMI was significantly correlated with intake of protein and lipid intake. However, no significant correlation was found between visceral fat area and intake of each nutrient. These results suggest that the presence of factors other than nutrient intake might be related to reduced visceral fat. Nutrient intake might be related to subcutaneous fat and muscle mass. Decreased subcutaneous fat is considered to result in decrease of leptin, which may enhance the susceptibility to MAC. Although the present study had a cross-sectional design and thus did not examine prognoses, a number of previous studies have shown that low BMI is a prognostic factor for MAC lung disease [4, 5]. Future tasks may include an investigation of possible effects of nutritional guidance on the prognosis and course of disease in patients with MAC lung disease.

Other characteristics from the nutritional aspect included a decreased serum prealbumin level, but albumin levels were within the normal range. No study has examined serum prealbumin level in patients with MAC lung disease. Decreased serum prealbumin level might be associated with the pathological conditions of patients with MAC lung disease; however, the underlying mechanisms are unclear.

There are several limitations to this study. First, this was a single-facility study with a small number of male patients. Moreover, patient data were not from their first visit (i.e., varying duration of time had elapsed after diagnosis in each case). Furthermore, the control data used for comparison were data of those who had participated in health checkups and thus may not necessarily be considered data of the general healthy population. Though we have compared visceral fat and nutritional status between patients with MAC lung disease and healthy controls, comparison between MAC lung and nonpathogen related inflammatory lung diseases would make the argument more compelling. In addition, a thin population would be a better control group rather than general population as we used in the study. Previous studies used general population as control [1, 2]. Though we have compared nutritional intake of patients with MAC lung disease with average intake among Japanese people determined based on the 2010 National Nutrition Survey, comparison with healthy controls might be more appropriate. A multicenter study is warranted, and it will be necessary to perform an investigation regarding visceral fat area and patient prognosis, as well as a prospective study in which dietary intervention is performed with a focus on lipid and protein intake.

In conclusion, this study revealed that patients with MAC lung disease have a low visceral fat area and low nutrient intake, although no significant correlation was found between them. This suggests that factors other than nutrient intake may underlie the reduced visceral fat area.

## Figures and Tables

**Table 1:** Baseline characteristics of patients with MAC lung disease (*n* = 103).

	Normal ranges	Total (mean ± SD)	Female (mean ± SD)	Male (mean ± SD)	*p*
Patient number		103	79	24	
Age (years)		72.3 ± 10.9	72.7 ± 11.1	71.2 ± 10.4	0.5628
Disease duration (months)		78.5 ± 65.0	78.7 ± 62.7	77.9 ± 73.6 months	0.9562
Smoking history		0/5^*∗*^/98	0/0/79	0/5^*∗*^/19	0.0005
Current/former/never					
Diabetes mellitus		9	4	5	0.0301
Emphysema		5^*∗∗*^	0	5^*∗∗*^	0.0005
Type of bacteria		*M. intracellulare 59* (57%)	*M. intracellulare 44* (56%)	*M. intracellulare 15* (63%)	
		*M. avium 35* (34%)	*M. avium 28* (35%)	*M. avium 7* (29%)	
		Both 9 (9%)	Both 7 (9%)	Both 2 (8%)	
Radiographic features					
FC dis.		26	14	12	0.0023
NB dis.		77	65	12	
Number of patients treated		71	53	18	0.4649
Height (cm)		154.0 ± 8.9	150.9 ± 7.1	164.2 ± 6.3	<0.0001
Weight (kg)		46.3 ± 9.3	43.8 ± 8.1	54.8 ± 7.8	<0.0001
BMI (kg/m^2^)		19.4 ± 2.9	19.2 ± 2.9	20.4 ± 2.7	0.0780
Visceral fat (cm^2^)		41.0 ± 33.9	35.7 ± 26.6	57.5 ± 47.4	0.0111
Waist (cm)		73.3 ± 8.4	72.5 ± 8.3	75.9 ± 8.5	0.0939
WBC (/*μ*L)	3100–8800	4968.6 ± 1559.1	4751.3 ± 1431.8	5675.0 ± 1769.1	0.0143
Lymphocytes (/*μ*L)	837–4136	1404.9 ± 526.2	1352.8 ± 456.8	1574.4 ± 691.6	0.0807
Alb (g/dL)	4.0–5.0	4.1 ± 0.3	4.1 ± 0.3	4.1 ± 0.3	0.3883
Ch-E (IU/L)	214–466	309.8 ± 74.0	309.5 ± 72.4	310.9 ± 80.6	0.9363
T-chol (mg/dL)	128–219	186.6 ± 28.0	190.9 ± 27.6	173.0 ± 25.1	0.0080
Transferrin (mg/dL)	190–320	212.0 ± 49.6	211.7 ± 43.1	213.1 ± 67.7	0.9013
Prealbumin (mg/dL)	22.0–40.0	16.9 ± 4.5	16.4 ± 4.4	18.4 ± 4.8	0.0662

Each parameter is displayed as mean ± standard deviation, except for patient number, comorbidity, type of bacteria, radiographic features, and number of patients treated.

FC dis.: fibrocavitary disease, NB dis.: nodular bronchiectatic disease, BMI: body mass index, WBC: white blood cell, Alb: albumin, Ch-E: cholinesterase, and T-chol: total cholesterol.

^*∗*^Three with FC dis. and two with NB dis.

^*∗∗*^Three with FC dis. and two with NB dis.

**Table 2:** Visceral fat, body mass index, and waist circumference of patients with MAC lung disease according to age (*n* = 103).

	Age (years)	Visceral fat (cm^2^)		*p* value	BMI (kg/m^2^)		*p* value	Waist (cm)		*p* value
		Patients Mean ± SD	Healthy persons Mean ± SD		Patients Mean ± SD	Healthy persons Mean ± SD		Patients Mean ± SD	Healthy persons Mean ± SD	
F	40~59	33.7 ± 28.0 (*n* = 13)	69.5 ± 35.4 (*n* = 99)	0.0007	20.4 ± 1.8 (*n* = 13)	23.4 ± 3.8 (*n* = 99)	0.0050	73.9 ± 7.7 (*n* = 13)	83.5 ± 8.8 (*n* = 99)	0.0003
	60~69	33.1 ± 18.7 (*n* = 14)	88.4 ± 46.6 (*n* = 43)	<0.0001	19.2 ± 2.9 (*n* = 17)	23.0 ± 3.2 (*n* = 43)	<0.0001	75.1 ± 6.3 (*n* = 14)	84.5 ± 8.9 (*n* = 43)	0.0006
	70~79	34.5 ± 30.5 (*n* = 20)	108.8 ± 44.1 (*n* = 32)	<0.0001	18.4 ± 2.6 (*n* = 22)	23.2 ± 2.7 (*n* = 32)	<0.0001	70.6 ± 8.2 (*n* = 20)	86.5 ± 7.3 (*n* = 32)	<0.0001
	80~	39.1 ± 27.4 (*n* = 25)	130.3 ± 51.8 (*n* = 8)	<0.0001	19.2 ± 3.5 (*n* = 27)	24.1 ± 2.7 (*n* = 8)	0.0009	71.8 ± 9.6 (*n* = 25)	89.6 ± 8.6 (*n* = 8)	<0.0001

M	50~59	57.5 ± 42.8 (*n* = 3)	114.6 ± 43.3 (*n* = 119)	0.0258	21.7 ± 2.5 (*n* = 4)	24.5 ± 2.6 (*n* = 119)	0.0435	77.8 ± 14.0 (*n* = 3)	87.7 ± 7.1 (*n* = 119)	0.0217
	60~69	75.8 ± 58.2 (*n* = 6)	126.8 ± 56.1 (*n* = 82)	0.0346	19.7 ± 3.6 (*n* = 6)	24.4 ± 2.7 (*n* = 82)	0.0001	76.4 ± 11.0 (*n* = 6)	88.4 ± 8.4 (*n* = 82)	0.0014
	70~79	39.7 ± 40.0 (*n* = 8)	126.8 ± 45.2 (*n* = 32)	<0.0001	20.4 ± 2.8 (*n* = 8)	24.1 ± 2.1 (*n* = 32)	0.0002	75.0 ± 6.6 (*n* = 8)	86.9 ± 5.8 (*n* = 32)	<0.0001
	80~	63.1 ± 50.5 (*n* = 6)	123.4 ± 50.5 (*n* = 10)	0.0368	20.1 ± 1.9 (*n* = 6)	23.8 ± 3.0 (*n* = 10)	0.0185	75.8 ± 7.1 (*n* = 6)	87.9 ± 9.0 (*n* = 10)	0.0141

Data are presented as mean ± standard deviation.

F: female, M: male, and BMI: body mass index.

**Table 3:** Correlation analyses between visceral fat area and each parameter in patients with MAC lung disease.

Parameter	Female		Male	
	*r*	*p* value	*r*	*p* value
Age	0.0325	0.7864	−0.0290	0.8955
Disease duration	0.0010	0.9935	0.3908	0.0652
BMI	0.5996	<0.0001	0.7534	<0.0001
Waist	0.7120	<0.0001	0.8425	<0.0001
WBC	0.0571	0.6364	0.5196	0.0110
Lymphocyte	0.0654	0.5882	0.6337	0.0012
Albumin	0.1447	0.2287	0.3879	0.0674
Ch-E	−0.0545	0.6519	0.2396	0.2709
T-chol	−0.1602	0.1819	0.1181	0.5916
Transferrin	0.0115	0.9241	−0.0547	0.8041
Prealbumin	0.1019	0.3977	0.5789	0.0038
CRP	−0.1876	0.1145	0.0905	0.6813

BMI: body mass index, WBC: white blood cell, Ch-E: cholinesterase, T-chol: total cholesterol, and CRP: C-reactive protein.

**Table 4:** Intake (%) of energy and each nutrient in patients with MAC lung disease.

	Total Mean ± SD
Energy (%)	86.1 ± 15.7
Protein (%)	82.4 ± 18.2
Lipid (%)	78.1 ± 21.8
Carbohydrate (%)	89.6 ± 19.2

Data are presented as mean ± standard deviation.

Intake (%) of energy and each nutrient was calculated by dividing the actual intake by the average intake among Japanese people determined based on the 2010 National Nutrition Survey × 100.

**Table 5:** Correlation analyses between body mass index and intake (%) of energy and each nutrient in patients with MAC lung disease.

Parameter	*r*	*p* value
Energy (%)	0.1961	0.0568
Protein (%)	0.2114	0.0397
Lipid (%)	0.2359	0.0214
Carbohydrate (%)	0.1092	0.2920

Intake (%) of energy and each nutrient was calculated by dividing the actual intake by the average intake among Japanese people determined based on the 2010 National Nutrition Survey × 100.

**Table 6:** Correlation analyses between visceral fat area and intake (%) of energy and each nutrient in patients with MAC lung disease.

Parameter	*r*	*p*-value
Energy (%)	−0.0242	0.8243
Protein (%)	0.0401	0.7123
Lipid (%)	−0.0103	0.9248
Carbohydrate (%)	−0.0025	0.9818

Intake (%) of energy and each nutrient was calculated by dividing the actual intake by the average intake among Japanese people determined based on the 2010 National Nutrition Survey × 100.

## Abbreviations

MAC: Mycobacterium avium complex
NTM: Nontuberculous mycobacteriosis
BMI: Body mass index
CT: Computed tomography
FC dis.: Fibrocavitary disease
NB dis.: Nodular bronchiectatic disease
SD: Standard deviation
WBC: White blood cell
Alb: Albumin
Ch-E: Cholinesterase
T-chol: Total cholesterol
CRP: C-reactive protein.
